# Acute Gastritis Supervening on Chronic

**Published:** 1830-07-01

**Authors:** 


### VIII.

ACUTE GASTRITIS SUPERVENING ON CHRONIC, ACCOMPANIED BY ARACHNITIS. Professor HORNER, of Pennsylvania.

"William C. aged 43, innkeeper, has used alcoholic drinks in excess for the last eight or ten years, and become much enfeebled from them. The last Summer he had a severe dysentery which lasted several weeks.

*Nov. 22d*, 1827.—I visited him for the first time.

*Habitude.* — Not much emaciated, skin pallid and temperate.

*Countenance.*—Dull and unmeaning.

*Intellectual Functions.* — Disposed to taciturnity, and dull in apprehension.

*Sensitive Apparatus.* — Hearing dull.

*Respiration* and *Circulation.* — Natural.

*Locomotive Apparatus.*—Very much enfeebled, scarcely able to walk.

*Digestive Apparatus.*—No appetite.

He did not complain of pain in any particular part. Ordered valerian tea.

*November 25th.*—I visited him again, and found him labouring under hallucinations, of which he was sensible; for he observed that though the figures were before his eyes, yet he knew they were deceptive. He complained also of pain in the epigastrium, and suffered from a retention of urine. The muscles of the abdomen were rigid, and drawn towards the spine. He had spent several nights without sleeping. Ordered opium two grains, and camphor one grain, to be made into a pill, and repeated every three hours till sleep be procured. Four of these pills produced the desired effect, and he slept soundly the following night.

The next day forty leeches were applied to the epigastrium with much advantage in diminishing the pain there, and two days afterwards a blister was put upon the same region.

*December 1st.*— His speech became suspended; great tenderness occurred in the abdomen, and the most excruciating pain in the lower extremities upon their being moved. His tongue became covered with a thick yellow coat, and his strength exceedingly prostrated. Volatile alkali was administered in a julep to the amount of five grains every two hours. He took several doses of it, and the next day I found that the moisture of the tongue had disappeared, and the yellow coat had dried up into a dark brown one. In the further progress of his treatment up to the day of his death, a mild cathartic was administered on three or four occasions, also a decoction of serpentaria and bark at intervals. His nourishment was wine whey, arrow root, and such light articles as he could be induced to swallow.

It was attempted twice to leech him on the head, but the leeches refused to bite; he was then cupped on the temples. He was also cupped along the spine, half a dozen cups on each side; and had mustard poultices applied to his ankles. He sunk gradually, and died this morning, December 7th, at four o'clock. From the day on which the retention of urine first occurred till the day of his death, the bladder continued paralytic, and an extremely fœtid, dark urine was daily brought off by the catheter. Also, for several days before death, he was incapable of moving the lower extremities, notwithstanding their extreme sensibility to the touch.

*Autopsy*. Twelve hours after death.

Head.—Very strong adhesion of dura mater to bone. In attempting to remove the latter, several drachms of serum were lost, which were supposed to come from beneath the tunica arachnoidea. The latter was turbid, and raised in vesications.

Blood-vessels of pia mater very turgid, as also those in the cerebrum; the latter on being cut into, bled freely, and much serum exuded from it. Cerebellum soft; adhesion between thalami unusually strong; a cluster of transparent vesicles on each side of plexus choroides; blood-vessels of velum very turgid. Spinal marrow, veins on surface very turgid; very great vascular fulness internally, giving a red pink colour along the roots of the anterior fasciculi of nerves where they came from within the medulla spinalis. Spinal marrow not so vascular along the roots of posterior fasciculi, but still having a superabundance of blood.

Thorax.—Ancient universal pleuritic adhesion on both sides; lungs healthy; heart healthy, its blood not coagulated.

Abdomen.—No peritoneal disease.

Stomach universally inflamed, and within of a deep pink colour, not coming from extravasation as in fever, but from the immense number and the fulness of its veins, which ran along the surface of the internal coat. At many places their capillaries were so numerous as to look at a little distance like small spots of extravasation, which, however, with the aid of a microscope were found to be congeries of very fine vessels. Near the cardiac orifice there was a round patch, two or two and a half inches in diameter, consisting of thickly interwoven veins, containing black blood, and looking as if they were varicose; they were on the internal surface of the mucous membrane. In the pyloric region were two [illegible]ddish slate-coloured patches, the indications of a chronic irritation there, and about twenty-four lines in diameter. Pylorus thickened; stomach small; scarcely any gas in the bowels.

Mucous coat of duodenum and jejunum inflamed to almost the same red colour with that of the stomach; ileum and colon of a bright pink colour internally; no ulceration of intestines; colon contained some well-elaborated fæces.

Liver common size, degenerated into a drab colour, hard, diminished vascularity; acini consisted in little hard scirrhus-like grains. The secretion of bile seemed to have been suspended, for the gall-bladder contained only a little black-coloured mucus.

Pancreas healthy; spleen healthy; kidneys healthy. Mucous coat of bladder inflamed, being injected with a network of veins, large and small, which were particularly abundant about the neck."*

We shall not attempt to decide on the priority of disease in the brain or

---

* American Journ. Med. Sciences, No. VIII.

the stomach, in the above case. That these two organs act and react on each other, is well known; but the extent of influence which the stomach is capable of exerting on the intellectual functions is not generally understood. The presence of air, acid, or both, in the stomach, duodenum, or upper intestines, has caused many acts of suicide—and every day causes intense mental suffering, without either patient or doctor being able to tell where the cause lies. Several instances have lately come within our knowledge, where the most intense despondency of mind and irritation of the feelings were almost instantly put to flight by a dose of Brandesh's liquor potassæ, a tea-spoonful of calcined magnesia, and some cinnamon water. The effects of these simple medicines are sometimes surprising. One gentleman assured us that after such a dose he discharged such quantities of flatus, upwards and downwards, that he was absolutely amazed where it could all come from. This was followed by a purgation which strong cathartics could not previously effect. The gloom of mind and irritability of temper disappeared with the discharges of flatus, and might be literally said to have "vanished into air." The same remedy produced similar phenomena again and again, not only in the individual alluded to, but in many others. This hint may prove useful to some of our brethren.

